# Incidence of Mild Cognitive Impairment and Dementia in Parkinson’s Disease: The Parkinson’s Disease Cognitive Impairment Study

**DOI:** 10.3389/fnagi.2019.00021

**Published:** 2019-02-08

**Authors:** Alessandra Nicoletti, Antonina Luca, Roberta Baschi, Calogero Edoardo Cicero, Giovanni Mostile, Marco Davì, Laura Pilati, Vincenzo Restivo, Mario Zappia, Roberto Monastero

**Affiliations:** ^1^Department of Medical and Surgical Sciences and Advanced Technologies “G.F. Ingrassia”, Section of Neurosciences, University of Catania, Catania, Italy; ^2^Department of Biomedicine, Neuroscience and Advanced Diagnostics, University of Palermo, Palermo, Italy; ^3^Department of Sciences for Health Promotion and Mother-Child Care, University of Palermo, Palermo, Italy

**Keywords:** mild cognitive impairment, dementia, Parkinson’s disease, incidence, neuropsychological assessment

## Abstract

**Background:** Cognitive impairment in Parkinson’s disease (PD) includes a spectrum varying from Mild Cognitive Impairment (PD-MCI) to PD Dementia (PDD). The main aim of the present study is to evaluate the incidence of PD-MCI, its rate of progression to dementia, and to identify demographic and clinical characteristics which predict cognitive impairment in PD patients.

**Methods:** PD patients from a large hospital-based cohort who underwent at least two comprehensive neuropsychological evaluations were retrospectively enrolled in the study. PD-MCI and PDD were diagnosed according to the Movement Disorder Society criteria. Incidence rates of PD-MCI and PDD were estimated. Clinical and demographic factors predicting PD-MCI and dementia were evaluated using Cox proportional hazard model.

**Results:** Out of 139 enrolled PD patients, 84 were classified with normal cognition (PD-NC), while 55 (39.6%) fulfilled the diagnosis of PD-MCI at baseline. At follow-up (mean follow-up 23.5 ± 10.3 months) 28 (33.3%) of the 84 PD-NC at baseline developed MCI and 4 (4.8%) converted to PDD. The incidence rate of PD-MCI was 184.0/1000 pyar (95% CI 124.7–262.3). At multivariate analysis a negative association between education and MCI development at follow-up was observed (HR 0.37, 95% CI 0.15–0.89; *p* = 0.03). The incidence rate of dementia was 24.3/1000 pyar (95% CI 7.7–58.5). Out of 55 PD-MCI patients at baseline, 14 (25.4%) converted to PDD, giving an incidence rate of 123.5/1000 pyar (95% CI 70.3–202.2). A five time increased risk of PDD was found in PD patients with MCI at baseline (RR 5.09, 95% CI 1.60–21.4).

**Conclusion:** Our study supports the relevant role of PD-MCI in predicting PDD and underlines the importance of education in reducing the risk of cognitive impairment.

## Introduction

Although Parkinson’s Disease (PD) has been classically considered a movement disorder, non-motor symptoms, such as cognitive impairment, represent very common features of the disease (Munhoz et al., 2015). Cognitive impairment encompasses a spectrum varying from Mild Cognitive Impairment (MCI) to dementia, and MCI is considered as an intermediate condition between “normal aging” and dementia (Petersen et al., 2001). This concept was originally used to early capture subjects at risk to develop Alzheimer’s disease (Petersen et al., 2001), and recently was extended and adapted to PD patients (Litvan et al., 2012). While a subtle cognitive impairment configuring MCI in PD (PD-MCI) could be diagnosed even in incident PD, a condition of overt dementia in PD (PDD) usually occurs in advanced stages with a prevalence close to 30% (Aarsland et al., 2017). Several risk factors have been associated with PDD occurrence, including old age at onset, long disease duration, severe motor impairment, and MCI (Hanagasi et al., 2017). Considering that PDD has a substantial negative effect on patient’s well-being and caregiver’s burden, the early detection of patients at risk to develop PDD deserves relevant prognostic and therapeutic implications (Aarsland et al., 2017).

To accurately identifying MCI in subjects with PD, in 2012 a task force of the Movement Disorder Society (MDS) proposed a standardized set of diagnostic criteria to be used both in daily clinical practice and research settings (Litvan et al., 2012). Depending on the comprehensiveness of neuropsychological testing, the MDS criteria provided two different diagnostic levels (i.e., Level I and Level II). Level I criteria allow for the diagnosis of PD-MCI through the administration of an “abbreviated” neuropsychological assessment, while Level II criteria recommend the administration of a “comprehensive” neuropsychological battery which permits the classification of PD-MCI into different subtypes, according to the cognitive domains impaired. Moreover, the identification of PD-MCI subtypes not only increases the diagnostic sensitivity but also allows hypothesizing MCI evolution and prognosis (Litvan et al., 2012). Studies carried out using these criteria have reported frequencies of PD-MCI ranging from 14.8 to 42.5% in patients with *newly diagnosed* PD (Yarnall et al., 2004; Poletti et al., 2012; Weintraub et al., 2018).

This study is part of The PArkinson’s disease COgnitive impairment Study (PACOS), an observational study involving two centers located in southern Italy (Sicily), aimed to evaluate frequency, clinical features and biomarkers associated with MCI in a large hospital-based cohort of PD patients (Baschi et al., 2018; Monastero et al., 2018). The PACOS cohort included 659 non-demented PD patients. In agreement with other studies, according to the MDS criteria, the prevalence of PD-MCI was 39.6% in the whole sample and 31.7% among *newly diagnosed* patients (disease duration ≤ 1 year). Amnestic MCI multidomain phenotype was the most frequent subtype recorded in 39.1% of the overall sample and 43.9% in *newly diagnosed* PD (Monastero et al., 2018).

Although several cross-sectional studies have evaluated the prevalence of cognitive impairment in PD, few longitudinal studies have assessed the incidence of PD-MCI according to the MDS criteria (Broeders et al., 2013; Pedersen et al., 2013, 2017; Domellof et al., 2015; Hobson and Meara, 2015; Pigott et al., 2015; Santangelo et al., 2015; Cholerton et al., 2018). Furthermore, only few studies adopted Level II MDS criteria for PD-MCI (Broeders et al., 2013; Domellof et al., 2015; Santangelo et al., 2015; Cholerton et al., 2018).

The aim of the present study was to evaluate the incidence of PD-MCI and PDD, the rate of progression from PD-MCI to PDD, and to identify demographic and clinical characteristics which predict cognitive impairment in a well-defined cohort of PD patients.

## Materials and Methods

### Study Population

Patients affected by PD diagnosed according to the Brain Bank criteria (Gibb and Lees, 1988) who attended the Neurologic Unit of the “Policlinico Vittorio Emanuele” in Catania and the Memory and Parkinson’s disease Center of the “Policlinico Paolo Giaccone” in Palermo, over a six-year period (2011–2016), were retrospectively enrolled in the PACOS cohort. The population included 659 non-demented PD subjects at baseline. All participants underwent a standard neurological workup, including a comprehensive neuropsychological assessment. Background and methods have been extensively reported elsewhere (Monastero et al., 2018).

Between 2014 and 2017 we retrospectively enrolled all PD patients who underwent at least two comprehensive neuropsychological evaluations (baseline and follow-up) during a period of maximum 48 months (between 12 and 48). All participants provided written informed consent prior to entering the study, which was approved by the Ethical Committee of the University Hospital of Palermo, P. Giaccone (approval number: 14:03/2018) and was in accordance with the Declaration of Helsinki.

### Clinical Assessment

All patients, at baseline and follow-up, underwent a standard neurological examination performed by neurologists experienced in movement disorders. Demographic, clinical and pharmacological data were collected from patient’s medical records. PD severity was evaluated with the Unified Parkinson Disease Rating Scale – Motor Evaluation (UPDRS-ME) and the Hoehn and Yahr (HY) scale. All patients were evaluated in “off” state. The clinical phenotype was attributed according to the classification in Tremor Dominant (TD), Postural Instability Gait Difficulty (PIGD) and Undetermined using scores from part II and III of UPDRS (Jankovic et al., 1990).

### Neuropsychological and Behavioral Assessment

All the enrolled patients, at baseline and follow-up, underwent a comprehensive neuropsychological assessment when in “on” state. Neuropsychological evaluations were performed by neurologists with a specific expertise in neuropsychology and dementia, and the same rater performed both baseline and follow-up assessments.

Patients underwent a Level I MDS criteria evaluation of global cognition using the following tests: the Mini Mental State Examination (MMSE) (Folstein et al., 1975), the Montreal Cognitive Assessment (MoCA) (Nasreddine et al., 2005), and the Frontal Assessment Battery (FAB) (Dubois et al., 2000).

According to MDS Level II criteria (Litvan et al., 2012), two tests for cognitive domains have been performed. The memory domain has been assessed with the Rey’s Auditory Verbal Learning Test (Carlesimo et al., 1996) and the Prose recall test with a delayed recall condition (Novelli et al., 1986a); the attention domain with the Stroop color-word test (Uttl and Graf, 1997) and the Trail Making Test part A (TMT-A) (Giovagnoli et al., 1996); the executive function domain with the Verbal fluency letter test (COWAT) (Novelli et al., 1986b) and the Colored Raven’s Progressive Matrices (Carlesimo et al., 1996); the visuo-spatial function domain with the Clock drawing test (CDT) (Shulman, 2000) and the Copy of figures (Carlesimo et al., 1996); lastly, the language domain has been assessed with the Aachener Aphasie Test-Naming item (Luzzatti et al., 1996) and the short version of the Token test (De Renzi and Vignolo, 1962).

For each test, details regarding administration procedures and Italian normative data for score adjustment (based on age, gender and education) were used. Neuropsychological performances were considered as impaired when the subject scored 2 standard deviation (SD) below normality cut-off values.

Mild cognitive impairment was diagnosed when patients scored below the cut-off values in at least two neuropsychological tests. MCI subtypes were defined as follows: amnestic MCI single domain (aMCIsd), when two of the memory tests were altered without impairment of other domains; non-amnestic MCI single domain (naMCIsd), when there were at least two tests altered within one single domain other than memory; amnestic MCI multi domain (aMCImd), when at least one memory test plus at least one test in any other domain were altered; non-amnestic MCI multiple domain (naMCImd), when two tests were altered in two different domains, without the involvement of the memory domain. The diagnosis of probable PDD was made according to the MDS criteria (Emre et al., 2007).

Functional independence was assessed using the Basic Activities of Daily Living (BADL) (Katz et al., 1963) and the Instrumental Activities of Daily Living (IADL) (Hughes et al., 1982). Lastly, Depression was evaluated using the Hamilton Depression Rating Scale, considering a cut-off scores > 9, as suggested by the MDS (Hamilton, 1960; Schrag et al., 2007).

### Statistical Analysis

Data were analyzed using STATA 12.1 software packages (StataCorp, College Station, TX, United States). Data cleaning was performed before the data analysis considering both range and consistence checks. Quantitative variables were described using mean and standard deviation. The difference between means and proportions was evaluated by the *t*-test and the Chi square test, respectively. In case of a not normal distribution, appropriate non-parametric tests were performed.

To calculate incidence rates of PD-MCI and PDD, we divided the number of cases with PD-MCI or PDD by the total number of person-years at risk during follow-up. We estimated person-years at risk (pyar) as the total follow-up time until PD-MCI or PDD. For incident PDD cases, we assigned time of dementia onset to the midpoint of the interval between assessments at which dementia was diagnosed. Because PD-MCI, in contrast to PDD, may be reversible or fluctuate over time, we set time of onset of incident PD-MCI to the exact date at which PD-MCI was first diagnosed. Incidence rates were also estimated considering only *newly diagnosed* patients (disease duration ≤ 1 year).

Kaplan–Meier survival analysis was carried out to estimate the cumulative proportion from normal cognition to any cognitive impairment (MCI or dementia) as well as the progression rate from MCI to dementia. The log-rank test was used to compare survival curves.

In order to identify possible predictors associated with the probability of progression from normal cognition to any cognitive impairment (MCI or Dementia) among the clinical and demographic characteristics, Cox proportional-hazards regression model was used for both the univariate and multivariate analyses. Variables with *p*-value < 0.1 at univariate analysis were included in the final multivariate Cox models. Schoenfeld residuals test was used for testing the proportional hazard. 95% confidence interval (CI), and *p*-value (two-tailed test, *a* = 0.05) were calculated. Analysis was also restricted to *newly diagnosed* PD patients.

Whenever variables were dichotomized or polychotomized, the cut-offs were derived from the pooled distribution of cases and control subjects (e.g., using the median value). To evaluate the role of dopaminergic therapy the levodopa equivalent daily dose (LED) was calculated for those patients taking dopamine agonists or levodopa in combination with dopamine agonists (Tomlinson et al., 2010).

## Results

The PACOS cohort consists of 659 non-demented PD patients (Monastero et al., 2018). Of 659 subjects, 139 PD patients (men 87, 62.6%) with a mean disease duration of 3.0 ± 2.8 years who underwent at least two neuropsychological evaluations between 12 and 48 months from 2014 to 2017 were enrolled in the present study. No significant differences in demographic and clinical characteristics were found between groups, apart from a borderline significant difference in disease duration between the two groups (see Supplementary Table S1). Of the 139 patients at baseline (first neuropsychological evaluation), 84 (60.4%) were classified as PD-NC, while 55 (39.6%) fulfilled the diagnosis of PD-MCI. Concerning the MCI subtypes, 4 (7.3%) patients had aMCIsd, 28 (50.9%) aMCImd, 12 (21.8%) naMCIsd and 11 (20.0%) naMCImd. Fifty-three (38.1%) of the 139 PD patients were *newly diagnosed* patients with a disease duration ≤ 1 year and of these 20 (37.7%) were classified as PD-MCI at the baseline evaluation. Baseline characteristics are shown in Table 1.

**Table 1:** Clinical and demographic characteristics at baseline.

	PD-NC *N* = 84	PD-MCI *N* = 55	Total *N* = 139	*p*-value
Men, *n* (%)	52 (61.9)	35 (63.6)	87 (62.6)	0.8
Age, years	64.4 ± 10.4	67.5 ± 7.4	65.7 ± 9.4	0.07
Age at onset, years	61.6 ± 11.0	64.5 ± 7.8	62.8 ± 10.0	0.09
Education, years	9.3 ± 4.4	8.3 ± 4.6	8.9 ± 4.6	0.2
UPDRS-ME score	25.4 ± 14.5	27.4 ± 11.9	26.2 ± 13.5	0.4
HY stage	1.9 ± 0.6	2.2 ± 0.7	2.0 ± 0.7	0.02
Disease duration, years	3.0 ± 2.9	3.0 ± 2.7	3.0 ± 2.8	0.9
Depression, *n* (%)	29 (34.5)	22 (40.0)	51 (36.7)	0.4
LED mg/day	437.2 ± 463.8	397.9 ± 408.8	421.8 ± 442.0	0.6
**Phenotype (%)**				
TD	32 (38.1)	11 (20.0)	43 (30.9)	/
PIGD	47 (55.9)	39 (70.9)	86 (61.9)	/
Mixed	5 (5.9)	5 (9.1)	10 (7.2)	0.07


*PD: Parkinson’s Disease; PD-NC: PD with Normal Cognition; PD-MCI: PD with Mild Cognitive Impairment; UPDRS-ME: Unified Parkinson’s Disease Rating Scale Motor Examination; HY: Hoehn and Yahr; LED: Levodopa Equivalent Daily Dose; TD: Tremor Dominant; PIGD: Postural Instability Gait Difficulty.*

### Incidence of MCI

Considering the 84 PD-NC at baseline, 28 (33.3%) fulfilled the diagnosis of PD-MCI, while 4 (4.8%) fulfilled the diagnosis of PDD at follow-up (mean follow-up time 23.5 ± 10.3 months). A slightly longer and borderline significant follow-up time was recorded among PD patients who developed MCI (25.7 ± 9.8 *versus* 21.3 ± 9.7 months; *p*-value 0.05), while a significantly longer follow-up was observed in the four patients who developed PDD (38.0 ± 9.6 months; *p*-value 0.004).

**FIGURE 1:**
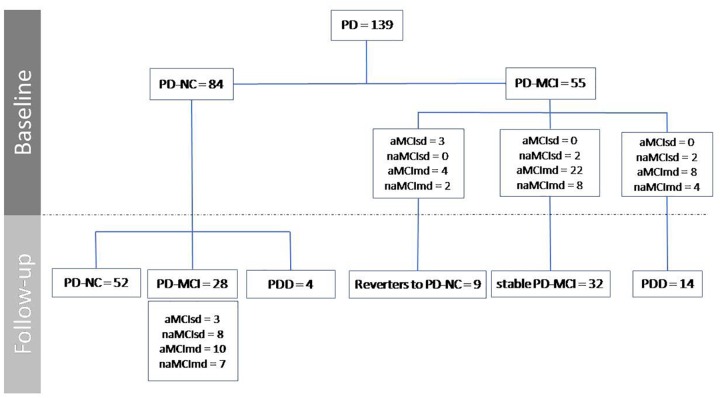
Progression of PD-NC and PD-MCI from baseline to follow-up.

Regarding the MCI subtypes, 3 (10.7%) out of the 28 patients developed an aMCIsd, 8 (28.6%) naMCIsd, 10 (35.7%) aMCImd and 7 (25.0%) naMCImd. The incidence rate of MCI among PD-NC at baseline was 184.0/1000 pyar (95% CI 124.7–262.3) (total person time at risk 152.2 years), without significant difference between sex [185.6/1000 pyar for men and 181.2/1000 pyar for women; relative risk (RR) 1.02, 95% CI 0.45–2.48; *p* = 0.5] (see Figure 1).

Out of the 84 PD-NC, 33 (39.3%) were *newly diagnosed* patients and of these 10 (30.3%) developed PD-MCI at follow-up. The incidence of MCI in *newly diagnosed* patients was 155.8/1000 pyar (95% CI 79.1–277.6) (total person time at risk 64.2 years). There was no significant difference between the incidence in the whole cohort and the incidence rate of *newly diagnosed* PD patients (RR 1.18, 95% CI 0.56–2.73; *p* = 0.3).

At univariate Cox proportional-hazards regression models, PD patients who developed MCI at follow-up were significantly older and with a lower level of education compared to those who preserved normal cognition (see Table 2). Multivariate analysis confirmed the strong protective effect of education in the development of MCI at follow-up with a Hazard Ratio (HR) of 0.37 for PD patients with more than 8 years of schooling (95% CI 0.15–0.89; *p* = 0.03) (see Table 2).

**Table 2:** Development of PD-MCI considering the 84 PD-NC at baseline.

			Univariate analysis			Multivariate analysis		
	PD-MCI *N* = 28	PD No-MCI *N* = 52	HR	95%CI	*p*-value	HR	95%CI	*p*-value
**Sex, Men (%)**	18 (64.3)	33 (63.4)	1.30	0.58–2.90	0.6	0.83	0.36–1.96	0.7
**Age, years**	68.5 ± 9.8	62.2 ± 10.3	1.04	1.00–1.09	0.04	1.04	0.99–1.08	0.07
Age ≤ 66 years	8 (28.6)	33 (63.5)	1	/	/			
Age > 66 years	20 (71.4)	19 (36.5)	2.81	1.24–6.41	0.01			
**Age at onset, years**	65.2 ± 10.8	59.6 ± 11.0	1.04	1.00–1.08	0.06			
Age at onset ≤ 50 years	2 (7.1)	10 (19.2)	1	/	/			
Age at onset > 50 years	26 (92.9)	42 (80.8)	2.10	0.49–8.99	0.3			
**UPDRS-ME**	24.4 ± 13.0	24.6 ± 14.2	0.98	0.95–1.00	0.2			
**HY stage**	2.1 ± 0.9	1.9 ± 0.5	1.01	0.73–1.40	0.9			
**Disease duration, years**	3.3 ± 3.1	2.6 ± 2.7	1.01	0.90–1.14	0.8			
**Education, years**	7.5 ± 4.8	10.5 ± 4.1	0.90	0.82–0.98	0.02			
Education ≤ 8 years	21 (75.0)	22 (42.3)	1	/	/	1	/	/
Education > 8 years	7 (25.0)	30 (57.7)	0.35	0.15–0.82	0.02	0.37	0.15–0.89	0.03
**LED mg/day**	480.8 ± 569.5	425.0 ± 415.6	1.00	0.99–1.00	0.5			
**Depression**	13 (46.4)	45 (31.8)	1.80	0.83–3.88	0.1			
**Phenotype**								
TD	11 (39.3)	19 (42.2)	1					
PIGD	15 (53.6)	24 (53.3)	0.75	0.34–1.65	0.5			
Mixed	2 (7.1)	2 (4.4)	3.50	0.72–16.9	0.1			


*Cox proportional-hazards regression models. PD: Parkinson’s Disease; PD-NC: PD with Normal Cognition; PD-MCI: PD with Mild Cognitive Impairment; HR: Hazard Ratio; UPDRS-ME: Unified Parkinson’s Disease Rating Scale Motor Examination; HY: Hoehn and Yahr; LED: Levodopa Equivalent Daily Dose; TD: Tremor Dominant; PIGD: Postural Instability Gait Difficulty.*

**FIGURE 2:**
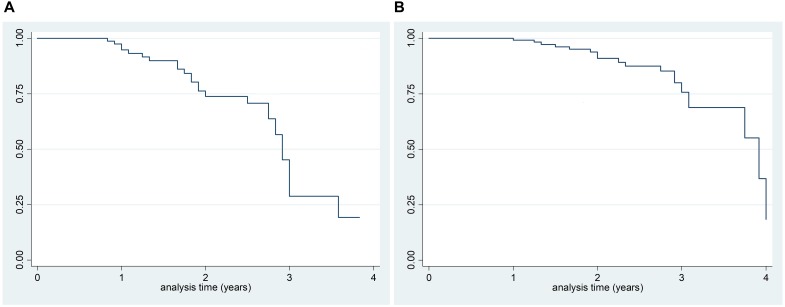
Kaplan–Meier survival analysis of PD-NC at baseline who developed PD-MCI at follow-up **(A)** and survival estimates of PD-MCI who developed PDD at follow-up **(B)**.

**FIGURE 3:**
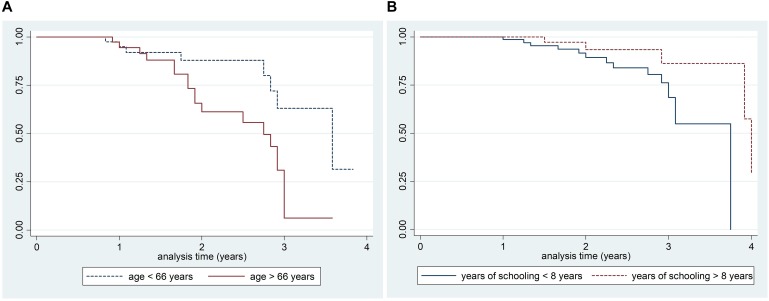
Kaplan–Meier survival estimates of PD-NC at baseline who developed PD-MCI at follow-up by age (**A**: Log rank test *p*-value 0.007) and education (**B**: Log rank test *p*-value 0.008).

According to Kaplan–Meier survival analysis, 94.8% (95% CI 91.3–99.8) of PD patients were free of MCI at 1 year of follow-up, 73.8% (95% CI 59.9–83.5) at 2 years of follow-up and 45.3% (95% CI 27.8–61.2) at 3 years as shown in Figure 2A. Close rates have been recorded when analysis was restricted to *newly diagnosed* patients [90.7% (95% CI 73.1–96.8) were free of MCI at 1 year, 76.7% (95% CI 54.5–89.0) at 2 years and 39.4% (95% CI 13.6–64.8) at 3 years]. A significant difference in survival curves, according to the log-rank test, has been found by age (>66 years and <66 years; *p* = 0.007) and education (years of schooling >8 years and <8 years; *p* = 0.008) as shown in Figure 3A,B.

Four of the 84 PD-NC developed dementia, giving an incidence rate of 24.3/1000 pyar (95% CI 7.7–58.5) (total person time at risk 164.8). These 4 patients presented a significantly higher UPDRS-ME (42.0 ± 21.5 *versus* 24.5 ± 13.7; *p* = 0.01) and a lower, although not significant, educational level (mean years of schooling 6.7 ± 4.5 *versus* 9.5 ± 3.5; *p* = 0.2).

### Incidence of PDD

Considering the entire sample of 139 PD patients, 18 fulfilled the diagnosis of PDD at follow-up (mean follow-up time 24.0 ± 10.2 months). A significantly longer follow-up time was recorded among PD patients who developed PDD (29.0 ± 11.1 months *versus* 23.3 ± 9.9 months; *p*-value 0.02). The incidence rate of PDD was 64.7/1000 pyar (95% CI 39.5–100.3) (total person-time at risk 278.2 years) with three times higher risk for men (70.1/1000 pyar for men and 56.1.0/1000 pyar for women; RR 3.2, 95% CI 1.11–10.4; *p* = 0.009).

Fifty-three (38.1%) of the 139 PD patients were *newly diagnosed* patients, of whom (11.3%) developed PDD with an incidence rate of 53.3/1000 pyar (95% CI 21.6–110.8) (total person time at risk 112.6 years). No significant difference has been recorded between the incidence in the whole cohort and incidence rate among the *newly diagnosed* PD patients (RR 1.21, 95% CI 0.46–3.73; *p* = 0.3).

At univariate analysis, Cox proportional-hazards regression model, PD patients who developed PDD at follow-up were significantly older, with a borderline but significantly higher UPDRS-ME score and a significantly lower education level compared to subjects who do not developed dementia (see Table 3). At univariate analysis the presence of MCI at baseline was the most important factor associated with the development of PDD (univariate HR 4.37, 95% CI 1.42–13.5; *p* = 0.01). This association was even stronger at multivariate analysis adjusting by age, sex *(a priori confounder)*, UPDRS-ME and education (HR 6.24, 95% CI 1.81–21.5; *p* = 0.004).

**Table 3:** Development of PDD.

			Univariate analysis			Multivariate analysis		
	PDD *N* = 18	No-PDD *N* = 121	HR	95%CI	*p*-value	HR	95%CI	*p*-value
**Sex, Men (%)**	12 (66.7)	75 (62.0)	1.21	0.44–3.37	0.7	2.42	0.76–7.74	0.1
**Age, years**	68.3 ± 8.4	65.3 ± 9.5	1.07	1.00–1.15	0.04	1.06	0.99–1.14	0.1
Age ≤ 67 years	7 (38.9)	62 (52.1)	1	/	/			
Age > 67 years	11 (61.1)	59 (48.8)	3.49	1.09–11.2	0.03			
**Age at onset, years**	64.9 ± 8.4	62.4 ± 10.1	1.05	0.99–1.12	0.1			
Age at onset ≤ 50 years	1 (5.6)	11 (10.0)	1	/	/			
Age at onset > 50 years	17 (94.4)	99 (90.0)	1.12	0.15–8.62	0.9			
**UPDRS-ME**	33.1 ± 16.5	25.1 ± 12.7	1.03	1.00–1.05	0.06	1.04	1.00–1.08	0.03
*UPDRS ≤ 25*	7 (38.9)	71 (58.7)	1	/	/			
*UPDRS > 26*	11 (61.1)	50 (41.3)	2.61	0.90–7.55	0.08			
**HY stage**	1.9 ± 0.6	2.1 ± 0.7	0.77	0.40–1.47	0.4			
**Disease duration, years**	3.4 ± 2.8	2.9 ± 2.8	1.07	0.92–1.25	0.3			
**Education, years**	7.4 ± 4.8	9.2 ± 4.5	0.88	0.78–0.99	0.04			
Education ≤ 8 years	13 (72.2)	69 (57.0)	1	/	/	1	/	/
Education > 8 years	5 (27.8)	52 (43.0)	0.28	0.08–1.03	0.06	0.36	0.09–1.52	0.2
**LED mg/day**	353.8 ± 298.4	432.1 ± 459.8	1.00	1.00–1.001	0.9			
**Cognition baseline**								
NC	4 (22.2)	80 (66.1)	1	/	/	1	/	/
MCI	14 (77.8)	41 (33.9)	4.37	1.42–13.5	0.01	6.24	1.81–21.5	0.004
**Depression**	9 (50.0)	42 (34.7)	1.28	0.50–3.24	0.6			
**Phenotype** (%)								
TD	6 (33.3)	37 (30.6)	1	/	/			
PIGD	12 (66.7)	74 (61.2)	1.01	0.38–2.76	0.9			
Mixed	0	10 (8.3)	/	/	/			


*Cox proportional-hazards regression models. PDD: Parkinson’s Disease Dementia; HR: Hazard Ratio; UPDRS-ME: Unified Parkinson’s Disease Rating Scale Motor Examination; HY: Hoehn and Yahr; LED: Levodopa Equivalent Daily Dose; NC: Normal Cognition; MCI: Mild Cognitive Impairment; TD: Tremor Dominant; PIGD: Postural Instability Gait Difficulty.*

**FIGURE 4:**
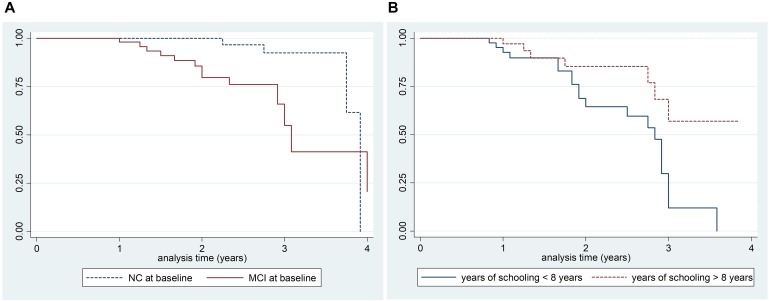
Kaplan–Meier survival estimates of patients who developed PDD at follow-up by the presence of MCI at follow-up (**A**: Log rank test *p*-value 0.004) and years of schooling (**B**: Log rank test *p*-value 0.005).

According to Kaplan–Meier survival analysis, 99.2% (95% CI 94.7–99.9) PD-MCI were free of dementia at 1 year of follow-up, 91.0% (95% CI 82.5–95.5) at 2 years, and 75.7% (95% CI 59.8–86.0) at 3 years (Figure 2B). A significant difference in survival curves was observed after stratifying for the presence of MCI at baseline (*p* = 0.005) and education (years of schooling > 8 years and ≤ 8 years; *p* = 0.04) as shown in Figure 4A,B. In particular, according to Kaplan–Meier survival analysis among PD-NC at baseline, the 100% were free of dementia at 1 and 2 years of follow-up, and 92.5% (95% CI 72.8–98.1) at 3 years. Considering PD-MCI patients, 98.1% (95% CI 87.1–99.7) were free of dementia at 1 year, 79.7% (95% CI 63.2–89.4) at 2 years, and only 55.0% (95% CI 28.2–75.4) at 3 years (Figure 4A).

Concerning the MCI subtypes, 14 out of the 18 patients who developed PDD had MCI at baseline; of these 12 (85.7%) were classified as MCI multi-domain (8 aMCImd and 4 naMCImd). A higher risk of developing PDD was recorded for those patients who had naMCImd at baseline with an adjusted HR of 21.1 (95% CI 3.89–114.7; *p* < 0.0001), followed by aMCImd (adjusted HR 8.70, 95% CI 2.01–38.0; *p* = 0.004). Overall, the risk of PDD was higher among patients with mdMCI, compared to those with sdMCI as shown in Table 4.

**Table 4:** PD-MCI subtypes, domains and risk of PDD.

			Multivariate analysis		
	PDD *N* = 18	No-PDD *N* = 121	HR	95%CI	*p*-value
**Model 1: MCI subtypes**					
Normal Cognition	4 (22.2)	80 (66.1)	1		
Amnestic MCI single domain	/	5 (4.13)	/	/	/
Non-amnestic MCI single domain	2 (11.1)	10 (8.3)	4.77	0.71-31.9	0.1
Amnestic MCI multi-domain	8 (44.4)	19 (15.7)	8.70	2.01-38.0	0.004
Non-amnestic MCI multi-domain	4 (22.2)	7 (5.8)	21.1	3.89-114.7	<0.0001
**Model 2: MCI single domain *versus* MCI multi-domain**					
Normal Cognition	4 (22.2)	80 (66.1)	1		
MCI single domain	2 (11.1)	15 (12.4)	2.51	0.39-15.8	0.3
MCI multiple domain	12 (66.7)	26 (21.4)	11.7	2.91-47.3	0.001
**Model 3: Amnestic MCI *versus* non-amnestic MCI**					
Normal Cognition	4 (22.2)	80 (66.1)	1		
Amnestic MCI	8 (44.4)	24 (19.8)	6.18	1.51-25.2	0.01
Non-amnestic MCI	6 (33.3)	17 (14.0)	8.58	2.11-15.8	0.003
**Impaired domain (at baseline)**^∗^					
Memory	8 (44.4)	33 (27.4)	1.88	0.62-5.64	0.3
Executive function	12 (66.7)	28 (23.1)	7.76	2.31-5.64	0.001
Attention	9 (50.0)	31 (25.6)	4.75	1.44-15.6	0.01
Visuo-spatial function	4 (22.2)	19 (15.7)	1.33	0.39-4.56	0.6
Language	1 (5.6)	1 (0.8)	38.2	3.31-441.3	0.004


*Cox proportional-hazards multivariate regression models. PD: Parkinson’s Disease; PD-MCI: PD with Mild Cognitive Impairment; PDD: PD with Dementia; HR: Hazard Ratio; HR adjusted by age sex, UPDRS-ME and education. ^∗^Impaired domain at baseline = at least one impaired test; for each domain the reference group is “not impaired.”*

Concerning the five different domains evaluated, in our sample a higher risk of dementia was recorded among PD patients presenting at least one impaired test in executive function at the baseline evaluation (HR 7.76, 95% CI 2.31–5.64; *p* = 0.001), followed by attention (HR 4.75, 95% CI 1.44–15.6; *p* = 0.01). Due to the presence of just two PD patients who presented an impaired language at baseline (one developed dementia at follow-up and one did not) with the consequent wide 95% CIs, the role of this domain is difficult to evaluate as shown in Table 4.

### Progression From MCI to PDD

Considering only the 55 patients with PD-MCI at baseline, 14 (25.4%) developed PDD at follow-up (mean follow-up time 24.7 ± 10.0 months), while 9 (16.3%) reverted to PD-NC. The incidence rate of PDD in patients with MCI at baseline was 123.5/1000 pyar (95% CI 70.3–202.2) (total person-time at risk 113.4), while the incidence rate of PDD among PD-NC at baseline was 24.3/1000 (95% CI 7.7–58.5), giving a RR of 5.09 (95% CI 1.60–21.4; *p* = 0.0009).

Of the 9 reverters, 3 (33.3%) were aMCIsd, 4 (44.4%) were aMCImd and 2 (22.2%) were naMCImd (see Figure 1). No significant differences regarding clinical and demographic characteristics at baseline between reverters and PD-MCI, irrespective of whether the patients developed PDD or not, were found.

## Discussion

In the present study we evaluated the incidence rate of PD-MCI and PDD and the risk of progression from PD-MCI to dementia in the PACOS cohort. At follow-up more than 33% of PD-NC at baseline developed MCI with an incidence rate of 184.0/1000 pyar, while more than 12% converted to PDD with an incidence rate of 24.3/1000 pyar. Conversely, the incidence rate of PDD among patients with MCI at baseline was 123.5/1000 pyar, giving a five time increased risk of developing dementia. Lastly, a significant negative association between education and PD-MCI was observed. PD-NC who converted to PD-MCI at follow-up were significantly less educated than non-converters. Moreover, the presence of MCI at baseline, in particular the naMCImd subtype, was strongly associated with PDD conversion, increasing the risk of dementia more than five times.

Mild cognitive impairment is considered an intermediate state between normal cognitive aging and early dementia. Several cross-sectional studies, the majority of which are multicenter studies, have been carried out to evaluate the prevalence of MCI during the last decade reporting ratios ranging from 18.9 to 35.2% (Foltynie et al., 2004; Aarsland et al., 2009). Differences in study designs, the definition of PD-MCI and the neuropsychological assessment adopted have greatly contributed to the wide variations in the reported estimates of PD-MCI. However, a high variability has also been reported across studies using the MDS criteria (Litvan et al., 2012) with MCI prevalence ranging from 20 to 41% (Broeders et al., 2013; Domellof et al., 2015; Pedersen et al., 2017; Weintraub et al., 2018). In agreement with these studies, in the PACOS cohort the prevalence of PD-MCI was 39.1% and MCI was associated with age and motor scores while a strong negative association was observed with educational level (Monastero et al., 2018).

Nonetheless, few prospective studies based on the MDS criteria for PD-MCI (Litvan et al., 2012) and PDD (Emre et al., 2007) have been performed until now, in order to evaluate the progression from normal cognition to MCI and the incidence of dementia (Broeders et al., 2013; Domellof et al., 2015; Pigott et al., 2015; Santangelo et al., 2015; Pedersen et al., 2017; Cholerton et al., 2018). Of these, four studies (Broeders et al., 2013; Domellof et al., 2015; Santangelo et al., 2015; Cholerton et al., 2018) have adopted Level II MDS criteria for the diagnosis of PD-MCI.

### Progression From Normal Cognition to MCI

Throughout the entire sample of 139 non-demented PD patients, the prevalence of PD-MCI at baseline was 44.6% and 39.2% considering only *newly diagnosed* patients; these rates were close to those reported for the whole PACOS cohort (Monastero et al., 2018), as well as those regarding other studies (Broeders et al., 2013; Domellof et al., 2015; Santangelo et al., 2015).

A lower frequency of MCI at baseline (20.2%) was reported in the Norwegian study (Pedersen et al., 2017), while the study by Cholerton et al. (2018) reported a higher prevalence of MCI. The latter result is probably due to the lower cut-off point used for the impairment on specific neuropsychological test (1 SD below normative data).

According to literature data, the most frequent type of MCI at baseline was the multiple domain (Santangelo et al., 2015), both amnestic and non-amnestic, representing the 49.1% and 20.0%, respectively.

At follow-up, 33.3% of PD-NC at the baseline developed MCI and considering only the *newly diagnosed* patients the frequency was 30.3%. These similar rates probably account for the short disease duration and mild motor impairment of the patients enrolled in the study. To the best of our knowledge, incidence rate of MCI among PD-NC was estimated only for the Norwegian study (Pedersen et al., 2017) where an incidence rate of 68.9/1000 pyar was recorded. This rate was lower with respect to our study, but it should be underlined that also a lower frequency of MCI at baseline was reported.

The results of survival analysis have demonstrated that approximately 5% of PD-NC developed MCI at 1 year, 26% at 2 years; these estimates are close to those reported by previous longitudinal studies conducted on PD-MCI, which had adopted MDS Level II criteria (Broeders et al., 2013; Santangelo et al., 2015; Pedersen et al., 2017). Nonetheless, approximately 55% of PD-NC developed MCI at 3 years, an estimate which is slightly higher than those reported in the literature (Broeders et al., 2013; Pigott et al., 2015; Pedersen et al., 2017). This difference can be in part explained by the enrolment of PD patients with a disease duration (mean disease duration: about 3 years) which is slightly longer than other cohorts (Broeders et al., 2013; Santangelo et al., 2015; Pedersen et al., 2017). At any rate when survival analysis was restricted to *newly diagnosed* patients close rates were found. Furthermore, it should be noted that PD patients enrolled in the present study had a lower educational level compared with other cohorts (Pigott et al., 2015; Santangelo et al., 2015; Pedersen et al., 2017) and this lower educational level probably contributed to a higher risk of developing MCI. Indeed, in agreement with these prospective studies (Santangelo et al., 2015; Pedersen et al., 2017), a strong protective effect of education was recorded with an almost 70% reduced risk of MCI in patients with more than 8 years of schooling.

### Progression From PD-NC and PD-MCI to Dementia

Incidence rate of PDD in the whole cohort (PD-NC and PD-MCI) was 64.7/1000 pyar and 53.3/1000 pyar among *newly diagnosed* patients. Again, the lack of differences between the whole sample and the *newly diagnosed* patients is probably due to the short disease duration of the entire sample. Only two prospective studies have evaluated the incidence rate of PDD reporting similar estimates. In particular, a close rate of 62.6/1000 pyar has been reported by Domellof et al. (2015), while a slightly lower rate of 38.1/1000 pyar was found in the Norwegian cohort (Pedersen et al., 2017). Nonetheless, comparison with this latter study is limited by the lower frequency of MCI reported at baseline (20.2%).

A significantly higher incidence rate of PDD was recorded among PD-MCI at baseline with respect to PD-NC, resulting in a five time increased risk of PDD among PD-MCI. Only two prospective studies based on MDS criteria, have evaluated the incidence rate of PDD among PD-NC patients and patients with MCI at baseline, both reporting very close results. In particular, similar rates were reported by Domellof et al. (2015) where the incidence rate of PDD was 18.8/1000 pyar among PD-NC and 142/1000 pyar among PD-MCI at baseline, leading to a 6.5 times increased risk of developing dementia among patients with MCI at baseline. Close results have also been reported in the Norwegian cohort, where incidence rate of PDD among PD patients presenting MCI at baseline was 120.8/1000 pyar, while the incidence in the whole cohort was 38.1/1000 pyar (Pedersen et al., 2017). A clear contribution of PD-MCI to the hazard of PDD was finally reported by an international study including longitudinal data from four different cohorts assessing cognition according to MDS Level II criteria. In this very recent study, only 6.4% of PD-NC developed dementia, while 50% of the PD-MCI group developed PDD (Hoogland et al., 2017). In agreement with previous studies (Domellof et al., 2015; Hoogland et al., 2017; Pedersen et al., 2017), the presence of MCI at baseline in the present study was the main predictor of PDD regardless of age, sex, educational level, and motor impairment as demonstrated by multivariate analysis.

In agreement with previous reports (Domellof et al., 2015; Pigott et al., 2015), in our cohort the risk of PDD was significantly associated with older age and motor impairment (borderline significant) at univariate analysis, and inversely associated with educational level. Of interest, at multivariate analysis and except for the presence of MCI at baseline, only motor impairment (UPDRS–ME) was still significantly associated with the risk of developing dementia.

In our study a three times increased risk of dementia among men (70.1/1000 pyar *versus* 56.1.0/1000 pyar) was found. The role of gender in the risk of cognitive impairment in PD is still debated, although several studies have suggested that male gender is a risk factor (Picillo et al., 2017). In a large multicenter case-control study, conducted in central-southern Italy, the association between PD and cognitive impairment was stronger among men compared to women (adjusted OR 5.44 for men and 2.82 for women) (Nicoletti et al., 2017). Nonetheless, and considering longitudinal studies, only a few studies have demonstrated a high risk of PDD among men (Pigott et al., 2015; Cholerton et al., 2018) and in particular in a recent study the principal predictive factor in the transition from PD-NC to PD-MCI or PDD was male sex with an OR of 4.47 (Cholerton et al., 2018).

Regarding the impact of specific MCI subtypes and cognitive domain in the progression from PD-NC to PD-MCI and dementia, naMCImd was the most important predictor of PDD after multivariate regression analysis, followed by aMCImd. To the best of our knowledge, none of the prospective studies based on MDS Level II criteria for the diagnosis of MCI have evaluated the role of the different MCI subtypes as a predictor for PDD development. Concerning specific cognitive domains (at least one impaired test at the baseline evaluation), executive functions and attention were strongly associated with the development of PDD. To date there has been no agreement regarding which type of impaired cognitive domain is a predictor of PDD. Indeed, according to the “dual syndrome hypothesis,” the impaired “cholinergic” visuo-spatial domain was more likely to evolve into later dementia, while the “dopaminergic” executive dysfunction was not (Kehagia et al., 2013). On the other hand, a recent study has confirmed the role of the cholinergic system in the maintenance of attention and executive functions as well (Lee et al., 2014). These neuroanatomical bases could support the role of the executive dysfunction as a significant predictor of PDD, as previously reported (Levy et al., 2002; Chung et al., 2017) and confirmed by our study.

The presence of at least one impaired test in the language domain in the present study was also strongly related to the development of PDD. However, it should be noted that only two subjects, both classified as PD-MCI at baseline, were impaired in the language domain, and only one developed PDD at follow-up. Accordingly, we believe that accuracy of this finding is questionable as confirmed by the wide CIs obtained. Furthermore, the frequency of the impairment of the language domain is generally rather low in subjects with PD-MCI (Santangelo et al., 2015).

According to previous reports, about 10% of patients classified as PD-MCI reverted to normal cognition over a period of years (Koepsell and Monsell, 2012). A possible explanation for this “inconstant” cognitive impairment could be: the effects of practice-related learning, normal fluctuation in cognition, depression, poor motivation, mild psychiatric symptoms, other medical condition or daytime sleepiness (Koepsell and Monsell, 2012). This complex and sometimes fluctuating course of cognitive impairment in PD increases the diagnostic uncertainly of the PD-MCI construct. In this study nine (16%) PD-MCI at baseline reverted to NC at follow-up. In non-PD patients, almost 40% MCI patients reverted to normal cognition during follow-up. Non-amnestic MCI and, more generically, single domain MCI have been reported to revert with high frequency (Roberts et al., 2014). Reversion in our cohort was not associated with a specific MCI subtype, although the small number of reverters does not provide accurate estimates.

Although the data presented in this paper relating to the incidence of PD-MCI and the progression from PD-MCI to PDD are close to those reported by other studies, comparisons should be interpreted with caution. Indeed, a wide variation in estimates has been reported by studies which used the MDS criteria. This variability in estimates should not only be due to the different study design: prevalent (Pigott et al., 2015) *versus* incident cases (Broeders et al., 2013; Domellof et al., 2015; Santangelo et al., 2015; Pedersen et al., 2017); population-based (Domellof et al., 2015; Pedersen et al., 2017) *versus* hospital-based (Broeders et al., 2013; Pigott et al., 2015; Santangelo et al., 2015), but also to the different level of the MDS criteria adopted [Level I criteria (Hobson and Meara, 2015; Pigott et al., 2015; Pedersen et al., 2017) *versus* Level II criteria (Broeders et al., 2013; Domellof et al., 2015; Santangelo et al., 2015; Cholerton et al., 2018)]. Furthermore, the different neuropsychological assessment adopted to evaluate cognitive impairment in PD may also account for the variability in estimates. Lastly, another relevant source of variability related to the MDS criteria, is the possibility of using different cut-off levels to consider a test as *impaired*. Indeed, the MDS has proposed a range of cut-off scores (1 and 2 Standard Deviations below normative data), but the choice of cut-offs levels impacts on prevalence estimates (Roberts et al., 2014).

The major strength of our study lies in the large cohort size of the PACOS study at baseline (Monastero et al., 2018), that allowed us to identify several non-demented PD patients, suitable to be re-evaluated at follow-up. Furthermore, a comprehensive neuropsychological assessment was used, fulfilling the requirements of Level II MDS criteria. Lastly, we used a cut-off score of 2 SD which produces reliable sensitivity and specificity levels, and its use in this field is, therefore, recommended (Goldman et al., 2013).

Nonetheless, several limits should be taken into account in interpreting our data. First, a possible selection bias cannot be excluded due to the hospital-based study design. Regarding other hospital-based cohorts (Foltynie et al., 2004; Aarsland et al., 2009; Hoogland et al., 2017), the presence of more severe cases attending the two hospital centers involved in the study cannot be excluded, and this may possibly have contributed to the high estimate of MCI at baseline. Nonetheless and, as previously reported (Monastero et al., 2018), the average HY score and the short disease duration recorded in the PACOS cohort have revealed a mild to moderate stage of disease. Second, although analyses were adjusted for major potential confounders, residual confounding (e.g., medical and neuropsychiatric comorbidity, the use of psychotropic drugs) cannot be excluded. Lastly, due to the small samples of some of the MCI subtypes in our longitudinal analysis, our results need to be confirmed and strengthened in larger cohorts.

In conclusion, despite the difference sources of variability across the few prospective studies conducted in PD-MCI based on MDS criteria, our data are in line with those previously reported. This supports the relevant role of MCI in the risk of developing dementia in PD patients and it underlines the importance of education in reducing the risk of cognitive impairment. Furthermore, the results of the present study may have relevant clinical and therapeutic implications. Indeed, considering the high risk of developing dementia, PD-MCI patients should be carefully monitored in order to benefit from both early pharmacological (Mamikonyan et al., 2015) and non-pharmacological interventions (Dibilio et al., 2017). Prospective data relating to large populations are required to confirm the risk of cognitive impairment in male subjects with PD, in addition to the specific cognitive phenotype, which is associated with the PD progression to MCI and dementia.

## Author Contributions

AN designed and conceptualized the study, analyzed and interpreted the data, and drafted the manuscript for intellectual content. AL collected and interpreted the data, and drafted the manuscript for intellectual content. RB and CEC collected the data and revised the manuscript for intellectual content. GM, MD, LP, VR and MZ interpreted the data and revised the manuscript for intellectual content. RM designed and conceptualized the study, interpreted the data, and drafted the manuscript for intellectual content. All authors approved the final manuscript.

## Conflict of Interest Statement

The authors declare that the research was conducted in the absence of any commercial or financial relationships that could be construed as a potential conflict of interest.
