# Supplementary material for: Incidence of Mild Cognitive Impairment and Dementia in Parkinson’s Disease: The Parkinson’s Disease Cognitive Impairment Study
Source: Front Aging Neurosci. 2019 Feb 8;11:21. doi: 10.3389/fnagi.2019.00021 (PMC6376919; doi:10.3389/fnagi.2019.00021)
Supplement: Supplementary file 1 [file Table_1.docx]

**Supplemental table**

**Table 1s. Clinical and demographic characteristics of the whole PACOS**

**sample (n=659) with patients included in the follow-up (n=139)**

|  | **Whole cohort**  **N=659** | **Included patients**  **N=139** | **p-value** | |
| --- | --- | --- | --- | --- |
| **Men, n (%)** | 379 (57.5) | 87 (62.6) | 0.3 |  |
| **Age, y** | 67.0 ± 9.7 | 65.7± 9.4 | 0.1 |  |
| **Age at onset, y** | 63.2 ± 10.5 | 62.8± 10.0 | 0.7 |  |
| **Education, y** | 8.1 ± 4.7 | 8.9 ± 4.6 | 0.06 |  |
| **UPDRS-ME score** | 25.8 ± 12.3 | 26.2 ± 13.5 | 0.7 |  |
| **HY stage** | 2.0 ± 0.6 | 2.0 ± 0.7 | 1.00 |  |
| **Disease duration, y** | 3.8 ± 4.6 | 3.0 ± 2.8 | 0.05 |  |
| **Depression, n (%)** | 265 (40.8) | 51 (36.7) | 0.4 |  |
| **MCI** | 261 (39.6) | 55 (39.6) | 0.99 |  |
| **Phenotype (%)** |  |  | 0.4 |  |
| TD | 196 (31.5) | 43 (30.9) |  |  |
| PIGD | 359 (57.7) | 86 (61.9) |  |  |
| Mixed | 67 (10.8) | 10 (7.2) |  | |

Abbreviations: UPDRS-ME: Unified Parkinson’s Disease Rating Scale Motor Examination; HY: Hoehn and Yahr; MCI: Mild Cognitive Impairment; TD: Tremor Dominant; PIGD: Postural Instability Gait Difficulty.
